# Development and post-Kasai procedure prognostic relevance of histological features for biliary atresia

**DOI:** 10.1186/s12887-023-04413-3

**Published:** 2023-11-22

**Authors:** Xiaodan Xu, Xueting Wang, Meiyun Ding, Yilin Zhao, Li Zhao, Linsheng Zhao, Mengdi Li, Fangyuan Zhao, Rongjuan Sun, Zhiru Wang, Ruifeng Zhang, Shujian Zhang, Liang Ge, Yan Sun, Jianghua Zhan

**Affiliations:** 1 Graduate College, Tianjin Medical University, Tianjin, 300070 China; 2 Department of General Surgery, Tianjin Children’s Hospital, LongYan Road 238, Beichen District, Tianjin, 300134 China; 3 Department of Pediatric Surgery, Xinjiang Yili Friendship Hospital, Yili, 835000 China; 4 Department of Pathology, Tianjin Children’s Hospital, Tianjin, 300134 China; 5 Department of Pediatric Surgery, Shandong Provincial Hospital Affiliated to Shandong First Medical University, Jinan, Shandong 250021 China

**Keywords:** Biliary atresia, Kasai procedure, Liver fibrosis, Staging system, Prognosis

## Abstract

**Objectives:**

To validate an appropriate evaluation method of liver fibrosis assessment based on the unique pathological features of biliary atresia (BA) that could well predict its prognosis.

**Methods:**

A total of 68 patients with BA who underwent Kasai procedure (KP) and an intraoperative liver biopsy, followed up from January 2019 to December 2021, were recruited in a retrospective analysis. Ishak, Metavir, and BA-specific staging systems in relation to outcomes were analyzed using logistic regression, COX proportional hazard regression, Kaplan-Meier analysis, etc.

**Results:**

Kaplan-Meier analysis determined a significant difference in native liver survival according to the BA-specific stage (*p* = 0.002). The ROC curve analysis for predicting prognosis showed that the AUC of BA-specific staging combined with iBALF and severe bile duct proliferation (BDP) (0.811, 95% CI: 0.710–0.913, *p* < 0.0001) was higher than BA-specific staging alone (0.755, 95% CI: 0.639–0.872, *p* < 0.001).

**Conclusions:**

The BA-specific staging system reflects the condition of the liver fibrosis, and its combination with iBALF and severe BDP helps to better evaluate the prognosis of patients with BA.

## Introduction

Biliary atresia (BA) is a severe neonatal disease characterized by biliary obliteration and progressive hepatic fibrosis [1]. If untreated, BA will inevitably lead to death from end-stage liver disease by age 2 years. Kasai procedure (KP) is the treatment of choice for restoring bile flow in patients with BA and some patients have achieved long-term native liver survival (NLS) through this procedure.

The severity of liver fibrosis at the time of KP affects long-term prognosis [2]. Currently, over 60% of BA patients require liver transplantation (LT) in the first year after KP, which indicates that the existing evaluation system for liver fibrosis of BA patients is insufficient for patient triage for performing KP or seeking LT promptly [1]. For some BA patients with severe fibrosis, early KP can’t reconstruct enough bile drainage, it may be much more beneficial to avoid the impact of KP and directly perform liver transplantation instead [3]. The major challenge for surgeons is to identify and select BA patients with good predicted prognosis for doing KP. This requires further research and a more valuable evaluation system.

The staging evaluation of liver fibrosis is a key step to improving the diagnosis and treatment of BA. Inflammatory damage to the intra- and extra-hepatic bile ducts with sclerosis and obliteration is a common histopathological feature of BA [4]. There are mature anatomical [5] and clinical classifications [6] of BA, but a successful and effective staging system has not been developed for several years. The Ishak [7] and Metavir [8] scores are based on the characteristics of liver fibrosis in adults with chronic hepatitis but have been generally used to grade the progression of liver fibrosis in BA.

However, BA features associated with prognosis are not considered in these scoring systems. Some studies have linked histopathological features of BA to the outcome, including the extent of histologic fibrosis [9, 10], hepatic inflammation [11], and bile duct proliferation (BDP) [12]. Further, the vast majority of BA patients have a rapid progression of liver fibrosis and some even have cirrhosis at first presentation, unlike chronic hepatitis. The use of adult scoring may not be a good guide to BA prognosis. Our team has found that approximately 1080–1860 BA patients are treated annually in 41 centers in mainland China [13]. To date the degree of liver fibrosis does not predict prognosis in most patients, so there is an urgent need to develop appropriate evaluation systems to guide the choice of post-operative treatment options.

Our study aimed to compare different histologic staging systems for BA that has hitherto been lacking by referring to classical staging systems. By comparing the various staging methods for BA, the selection of the best option for assessing prognosis can be used by the surgeon to design an appropriate treatment plan for BA patients.

## Methods

### Study population

The study was conducted at the Department of Pediatric Surgery and Pathology, Tianjin Children’s Hospital, China. Patients were identified retrospectively by reviewing the clinical and pathology databases of subjects diagnosed with BA from January 2019 to December 2021. Diagnosis of BA was confirmed by intraoperative cholangiography findings and histological examination of liver. Study inclusion criteria were: (1) diagnosis of type III BA, (2) completed KP, (3) available clinical details and follow-up data after KP, (4) available complete liver biopsies taken at KP. Study exclusion criteria were: (1) type I or II BA, (2) a final diagnosis other than BA, (3) missing or inadequate data. A total of 87 BA patients were admitted during this period, excluding 1 patient with type I BA, 16 patients who did not undergo KP, 2 patients with inadequate biopsy samples, 68 patients remained for analysis.

Our Kasai procedure followed the Guidelines for diagnosing & treating biliary atresia (2018 Edition) [14]. Dissect the cone-shaped fibrous plate in the hepatic hilum, ligate the branches from portal vein to the fibrous plate, and transect the fibrous plate at the level of the liver capsule without damaging the liver parenchyma. The jejunum was dissected 15–25 cm distal to the ligament of Treitz, and the jejunum was preserved 30–45 cm from the biliary branch of the jejunum to perform an end-side anastomosis.

The data collected included demographics, biochemical tests and follow-up data. The demographic information included sex and age at the time of KP. The biochemical parameters included total bilirubin (TB), direct bilirubin (DB), aspartate aminotransferase (AST), alanine aminotransferase (ALT), γ-glutamyl-transpeptidase (GGT), alkaline phosphatase (ALP), total bile acid (TBA), albumin, platelet count, AST to platelet ratio index (APRI) and infant BA liver fibrosis (iBALF). APRI score = AST level (U/L)/upper normal × 100/platelet count (10^9^/L) [15]. iBALF score = 8 + 1.185 × Log_e_ [TB (mg/dl)] − 1.882 × Log_e_[platelet count (10^9^/L)] + 1.093 × Log_e_ [age (days)] [16]. Follow-up data included cholangitis, jaundice clearance (JC) and native liver survival (NLS). This study was conducted in accordance with the declaration of Helsinki. This study was approved and implemented by the ethics committees of Tianjin Children’s Hospital (2022-SYYJCYJ-008) and all patients provided consent for participation in medical research. Informed consent was obtained from all subjects and/or their legal guardian(s).

### Histology assessment

Wedge biopsy specimens of the liver were collected intraoperatively, fixed in formalin and embedded in paraffin to make four-micrometer-thick tissue sections. Each histological section included at least ten hepatic lobules and was routinely stained using hematoxylin-eosin (HE) and cytokeratin 19 (CK19). All sections were assessed for fibrosis grades and other histological features.

Fibrosis was graded using the Ishak score [7], Metavir score [8], and BA-specific score for BA. The Ishak scoring system classified liver fibrosis into six stages, stage 1, fibrous expansion of some portal area, with or without short fibrous septa; stage 2, fibrous expansion of most portal area, with or without short fibrous septa; stage 3, fibrous expansion of most portal area with occasional portal to portal (P-P) bridging; stage 4, fibrous expansion of portal area with marked bridging, (portal to portal (P-P) as well as portal to central (P-C)); stage 5, marked bridging (P-P and/or P-C) with occasional nodules (incomplete cirrhosis); stage 6, cirrhosis, probable or definite. The Metavir scoring system classified liver fibrosis into four stages: stage 1, portal fibrosis without septa; stage 2, portal fibrosis with rare septa; stage 3, numerous septa without cirrhosis; stage 4, cirrhosis with nodule formation. The BA-specific scoring system classified liver fibrosis into four stages: stage 1, fibrous expansion in portal tracts, with or without short fibrous septa; stage 2, short fibrous septa in portal area, bridging fibrosis involving < 50% of portal tracts; stage 3, bridging fibrosis with > 50% of portal tracts involved, signs of early cirrhosis with occasional nodules; stage 4, nodule formation and cirrhosis.

**Fig. 1:**
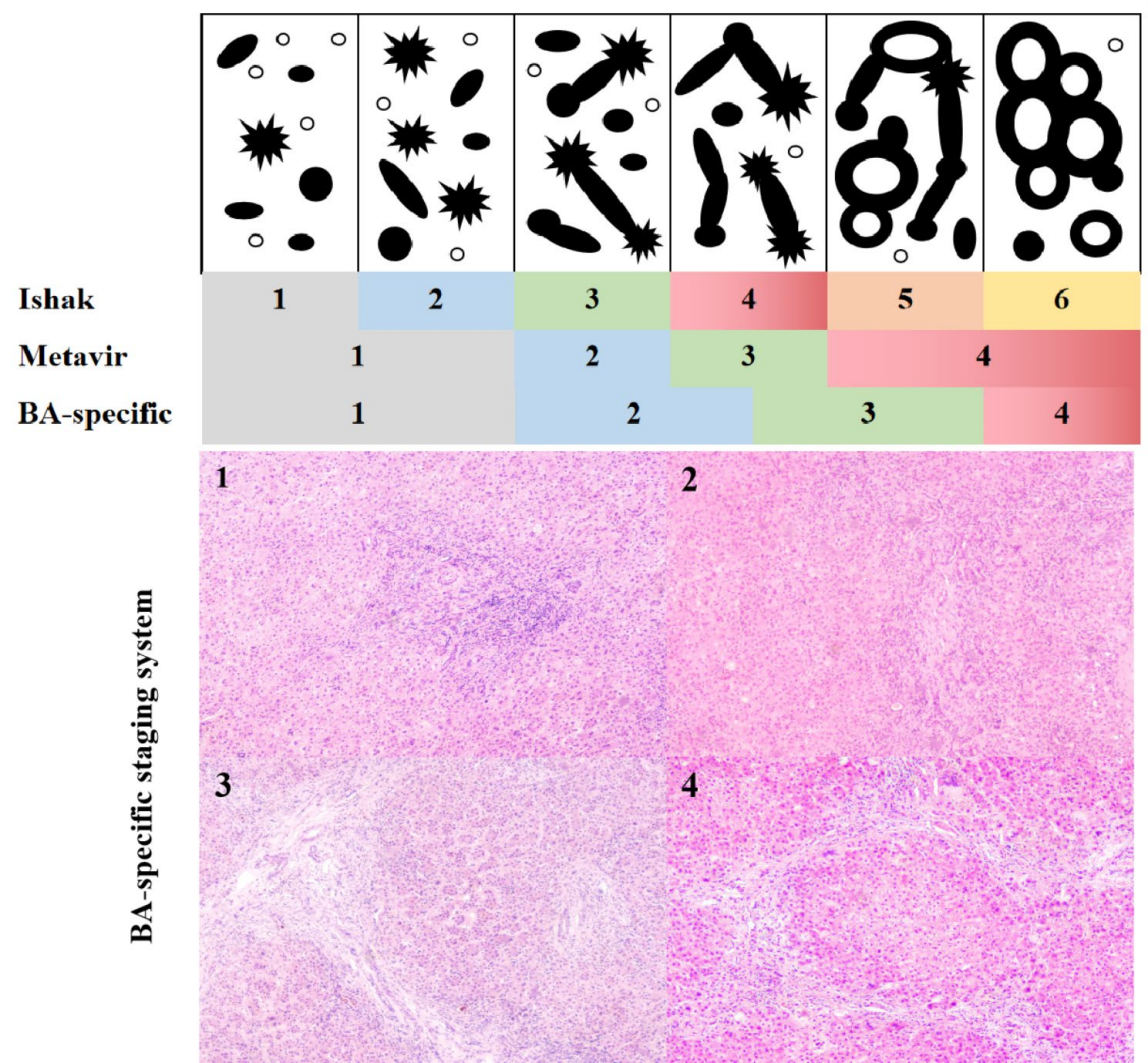
BA-specific staging system. Diagrammatic representation of the six stages (1–6) of the Ishak fibrosis score compared to the four stages (1–4) of the Metavir fibrosis score and the four stages (1–4) of the BA-specific fibrosis score. Dark circles indicate portal areas, open circles indicate the walls of central veins, spike-like septa indicate fibrous expansion of portal tracts. 1–4 corresponding to BA-specific stage 1–4 respectively (40x total magnification)

The BA-specific fibrosis stage was based on (1) characteristic pathomorphological features of BA [14, 17], (2) previously reported a three grade staging systems for BA [10, 18], and (3) inter-observer agreement as documented in the literature [10] or according to the findings of this study (Fig. 1). The use of a four-stage scoring method like Metavir’s reflected the changes in fibrosis, while the inclusion of fibrous septa like in Ishak was to better determine the broadening extent of the portal area in BA [19].

The study also selected the histological features that might be strongly associated with the outcome, including the extent of the portal and periportal inflammation, cholestasis, and bile duct proliferation (BDP) [20, 21] in liver biopsies. Portal and periportal inflammation was classified into three grades: grade 1 (mild), inflammatory cells seen in < 1/3 of the portal tracts; grade 2 (Moderate), inflammatory cells seen between ≥ 1/3 to < 2/3 of the portal tracts; grade 3 (Severe), inflammatory cells dense and present in ≥ 2/3 of portal tracts. Cholestasis was classified into three grades: grade 1 (mild), bile accumulation in the canalicular or centrilobular hepatocytes; grade 2 (Moderate), bile accumulation in the centrilobular and periportal hepatocytes or in the portal tract; grade 3 (Severe), presence of bile infarcts. Bile duct proliferation was classified into three grades: grade 1 (mild), 5–9 bile ducts per portal tract; grade 2 (Moderate), ≥ 10 bile ducts per portal tract; grade 3 (Severe), ≥ 10 bile ducts per portal tract and the ducts are elongated, attenuated and angulated.

All slides were reviewed by two senior pathologists and the difference of opinion was solved by a common consensus. The observers were unaware of the clinical data. Inter-observer kappa values for different pathological parameters were 0.674–0.836. The kappa values for Ishak, Metavir and BA-specific staging were 0.674, 0.812 and 0.787. The kappa values for portal inflammation, intrahepatic cholestasis and ductal proliferation grades were 0.699, 0.747 and 0.836.

### Follow-up evaluation

Patients were followed-up monthly for the first 6 months after KP and every 3–12 months thereafter, with the final follow-up visit in June 2022. Follow-up methods included outpatient, telephone and WeChat. Local patients will contact us by phone and visit outpatient regularly to review the indicators. Patients from other places will receive timely follow-up examinations in the local area and send photos of the examinations to us via WeChat. At each visit, general conditions (body temperature, skin color, stool color), medical history (cholangitis, JC, NLS) and physical examination were performed along with routine laboratory tests. Observed follow-up outcomes included cholangitis, JC and NLS. Cholangitis was defined as fever > 38℃ or elevated inflammatory markers and evidence of cholestasis or abnormal liver function tests following Tokyo guidelines [22]. Good JC was defined as TB postoperative levels below 20µmol/L at 6 months post-surgery [23]. Poor JC was defined as TB levels higher than 20µmol/L at 6 months post-surgery or at the time of death or liver transplantation within 6 months post-surgery. To analyze factors associated with outcome at follow-up time, patients with mortality or liver transplantation during follow-up were screened and counted as events; patients with non-liver-related deaths and those who survived with native liver were counted as event-free. Loss to follow-up or deaths due to non-liver-related events are censored data, using all information up to the time of censorship for further analysis. Clinical data, JC and NLS were evaluated in relation to liver fibrosis score.

### Statistical analysis

Results were presented as number (percentage), mean ± standard deviation (SD), or median (interquartile range [IQR]) depending on the variable data characteristics. Study group comparison was carried out using the Chi-squared test or Fisher’s exact test, Student’s t-test and Mann–Whitney-Wilcoxon-test. The kappa consistency test was calculated to measure the interobserver agreement. The correlation of grades in fibrosis with other parameters was tested using Spearman’s test. Univariate and multivariate logistic regression analyses were performed to calculate odds ratio (OR) estimates for outcomes. The cumulative incidence of events and event-free during the follow-up evaluation were calculated using Kaplan–Meier analysis and compared by log-rank testing. Univariate and multivariate hazard ratio (HR) estimates for outcomes were calculated by COX proportional hazard regression analysis. The predictive performance of the three different liver fibrosis staging methods on outcome was assessed by receiver operating characteristics (ROC) curves. The area under the receiver ROC curve (AUC) was calculated. *P*-values < 0.05 were considered statistically significant. All of the data management and statistical analysis were performed using SPSS 25.0 and GraphPad Prism 8.0.1.

## Results

### Baseline characteristics

**Fig. 2:**
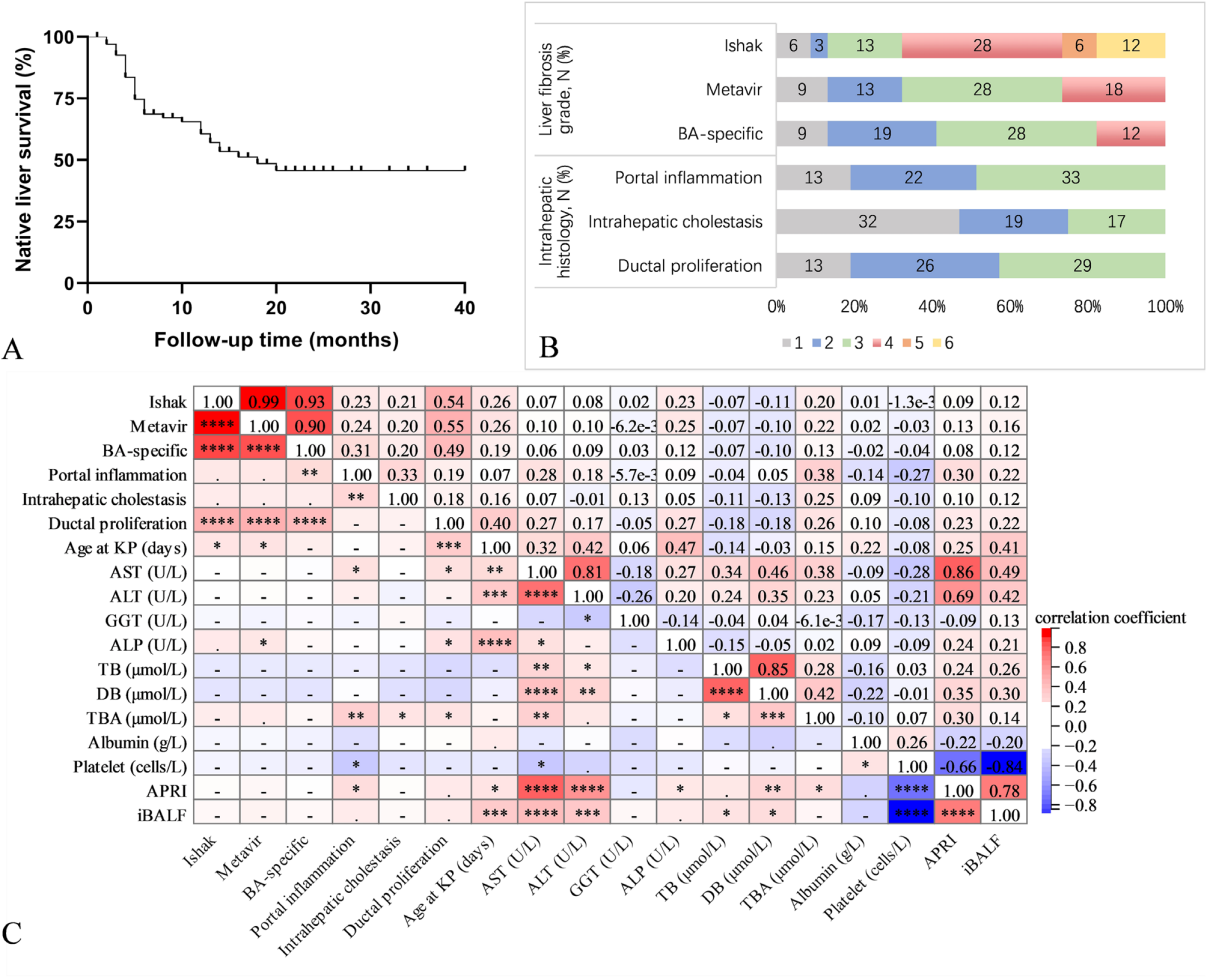
(**A**) Overall native liver survival curve (**B**) Liver biopsy characteristics of the patient population (N = 68) (1–6 scores represent histologic staging and grading). (**C**) Correlation of liver biopsy characteristics with other parameters studied. The color of the box represented the strength of the correlation, the redder the color, the stronger the positive correlation, and the bluer the color, the stronger the negative correlation. * indicates *p* < 0.05, ** indicates *p* < 0.01,*** indicates *p* < 0.001, **** indicates *p* < 0.0001

A total of 68 infants met the inclusion and exclusion criteria and followed at our center during the study period. The patients’ clinical, and laboratory characteristics at the time of KP are summarized in Table 1. Female patients constituted 66.2% of the cases. The mean age at the time of the surgical intervention was 62.5 ± 21.3 days. The median follow-up time was 13 (5–20) months. 6 (8.8%) patients were lost to follow-up evaluation. It was estimated that 1-year native liver survival rate was 60.6% (Fig. 2A). By the end of follow-up, a total of 28 (41.2%) patients had undergone liver transplantation, and 5 (7.4%) patients had died of liver failure. Most death/liver transplantation events occurred within 1 year after KP. The liver biopsy characteristics of the patient population were described in Fig. 2B. Both Metavir and BA-specific staging had the highest proportion of 28 (41.2%) cases at stage 3, while Ishak staging had the highest proportion of 28 (41.2%) cases at stage 4.

**Table 1:** Characteristics of the study population (N = 68)

Characteristics	Total
Age at KP (days)	62.5 ± 21.3
Female (%)	45 (66.2%)
AST (U/L)	181 (132–310)
ALT (U/L)	112 (75–160)
GGT (U/L)	397 (242–639)
ALP (U/L)	594.2 ± 207.9
TB (µmol/L)	177.1 ± 48.6
DB (µmol/L)	138.0 ± 40.0
TBA (µmol/L)	112 (92–138)
Albumin (g/L)	39.5 (37.4–42.4)
Platelet (cells/L)	418 ± 150
APRI	1.46 (0.74–2.67)
iBALF	4.04 (3.33–4.56)
Ishak (stage)	4 (3–5)
Metavir (stage)	3 (2–4)
BA-specific (stage)	3 (2–3)
Portal inflammation (grade)	2 (2–3)
Intrahepatic cholestasis (grade)	2 (1–3)
Ductal proliferation (grade)	2 (2–3)
Jaundice clearance (%)	23 (33.8%)
Cholangitis (%)	43 (61.4%)
Follow up (months)	13 (5–20)
1-year native liver survival (%)	60.6%

Measurement data with normal distribution were presented as means ± standard deviation (SD). Non-normal distribution data were presented as median (interquartile range [IQR]). Qualitative data were presented as number (percentage)

Moreover, there was a correlation between fibrosis grade and the histological features (Fig. 2C). Portal inflammation grade correlated significantly with BA-specific stage (r = 0.31; *p* < 0.01). Ductal proliferation correlated significantly with three liver fibrosis staging, including Ishak (r = 0.54; *p* < 0.0001), Metavir (r = 0.55; *p* < 0.0001), and BA-specific (r = 0.49; *p* < 0.0001). In contrast, intrahepatic cholestasis did not correlate with any of the three liver fibrosis staging.

### Association of liver histology with jaundice clearance

**Fig. 3:**
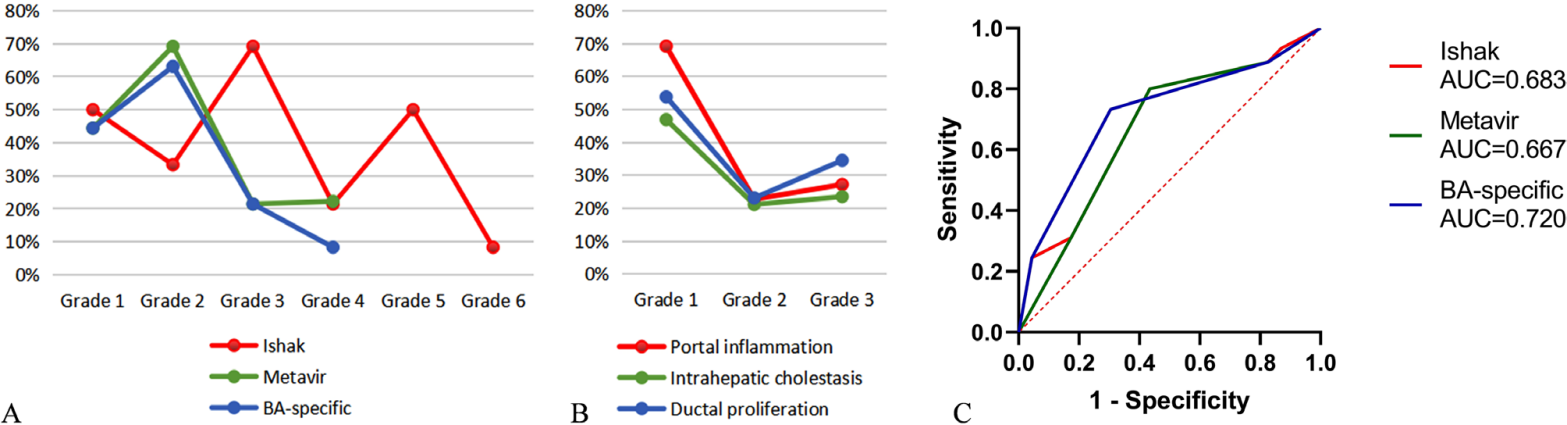
(**A**) Change in jaundice clearance with each stage of Ishak, Metavir, BA-specific. (**B**) Change in jaundice clearance with each grade of portal inflammation, intrahepatic cholestasis, ductal proliferation. (**C**) The ROC curve of staging systems associated with jaundice clearance, including Ishak, Metavir and BA-specific.

The overall trend of JC decreased as the degree of liver fibrosis and pathological features increased (Fig. 3A, B). Following a univariate analysis, we discovered that Ishak/Metavir staging (OR 5.2, 95% CI 1.73–15.64, *p* = 0.002), and BA-specific staging (OR 6.29, 95% CI 2.08–19.02, *p* = 0.001) may be independent indicators of poor JC (Table 2). Notably, 73.3% of patients with poor JC were stage 3–4 according to BA-specific staging. There was no significant association between JC and other histopathological features or between the various laboratory data. However, Metavir/Ishak staging (*p* = 0.953), BA-specific staging (*p* = 0.06), and age at KP (*p* = 0.211) had no significant effect on JC when multivariate analyses were performed (Table 2). The ROC curve analysis for predicting JC using the liver fibrosis staging systems revealed an AUC of 0.667 (95% CI: 0.526–0.807, *p* = 0.025) for Metavir, 0.683 (95% CI: 0.548–0.817, *p* = 0.014) for Ishak, 0.720 (95% CI: 0.595–0.846, *p* = 0.003) for BA-specific (Fig. 3C).

**Table 2:** Predictors of poor jaundice clearance

	Good JC (n = 23)	Poor JC (n = 45)	Odd ratio	95% confidence interval	*p* value
**Univariate analysis**					
Ishak/Metavir					
1–3/1–2 (n = 22)	13	9	5.2	1.73–15.64	**0.002**
4–6/3–4 (n = 46)	10	36			
BA-specific			6.29	2.08–19.02	**0.001**
1–2 (n = 28)	16	12			
3–4 (n = 40)	7	33			
Portal inflammation			1.78	0.64–4.94	0.268
Mild and moderate (n = 35)	14	21			
Severe (n = 33)	9	24			
Intrahepatic cholestasis			1.93	0.55–6.78	0.3
Mild and moderate (n = 51)	19	32			
Severe (n = 17)	4	13			
Ductal proliferation			0.95	0.34–2.62	0.92
Mild and moderate (n = 39)	13	26			
Severe (n = 29)	10	19			
Age at PE			2.57	0.91–7.28	0.073
< 60 (n = 34)	15	19			
≥ 60 (n = 34)	8	26			
Cholangitis			1.17	0.41–3.28	0.77
No (n = 25)	9	16			
Yes (n = 43)	14	29			
**Multivariate analysis**					
Ishak/Metavir			0.94	0.13–6.64	0.953
BA-specific			6.05	0.93–39.44	0.060
Age at PE			2.16	0.65–7.19	0.211

The data is tabulated for number of patients according to good or poor jaundice clearance (JC) by those with or without cholangitis; age at Kasai of ≥ 60 days or < 60 days; Ishak/Metavir stage 1–3/1–2 vs. 4–6/3–4; BA-specific stage 1–2 vs. 3–4; portal inflammation, intrahepatic cholestasis, and ductal proliferation severe vs. mild/moderate. Univariate and multivariate analysis showing odd ratio and 95% confidence interval for poor JC. Results with *p*-values < 0.1 were subjected to multivariate analysis after univariate analysis. *P*-values < 0.05 were considered statistically significant

### Association of liver histology with native liver survival

**Fig. 4:**
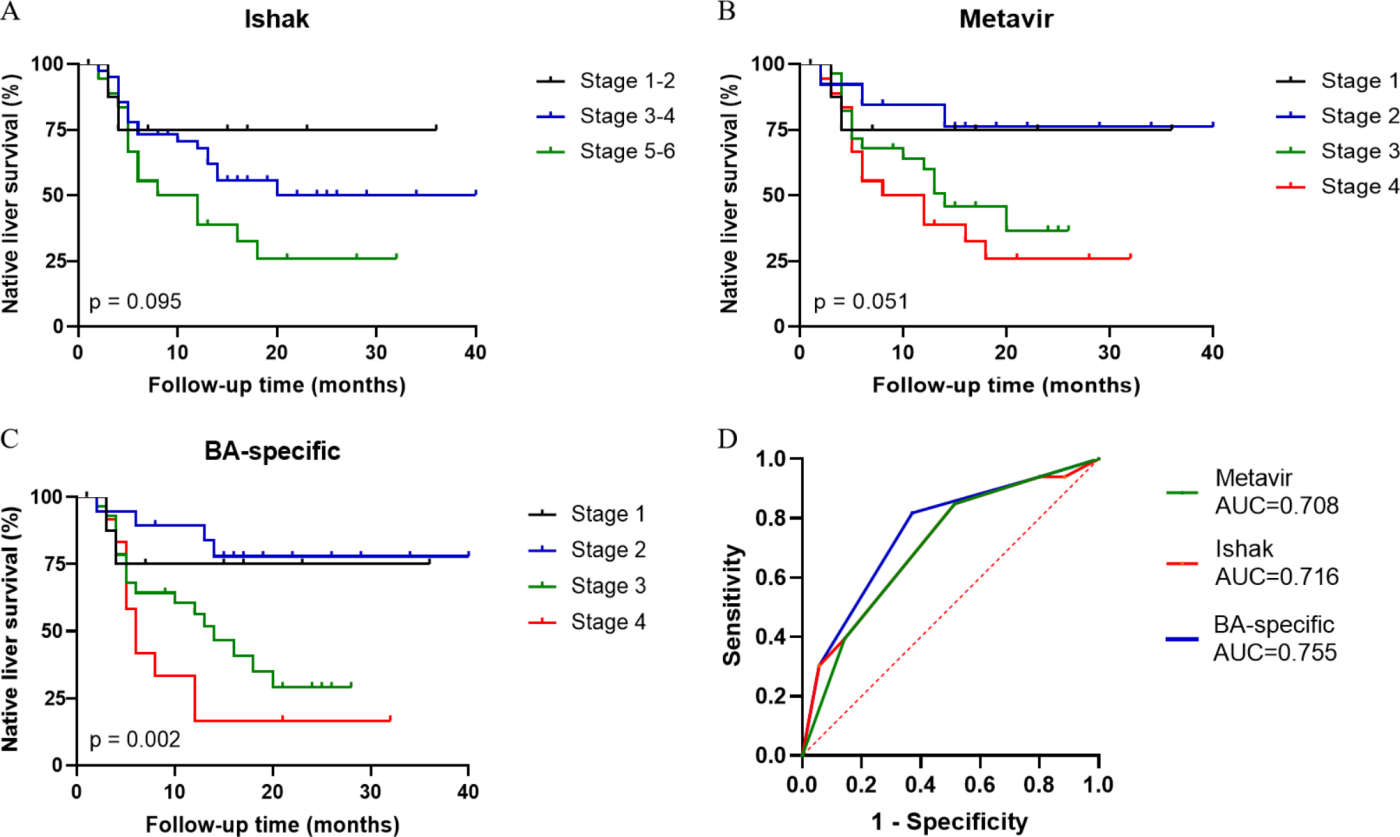
Kaplan-Meier plots of native liver survival by Ishak (**A**), Metavir (**B**) and BA-specific (**C**) stages. (**D**) The ROC curve of staging systems associated with native liver survival, including Ishak, Metavir and BA-specific.

Thirty-three patients (48.5%) had mortality/liver transplantation and liver-related events during follow-up evaluation. The cumulative probability of events through the different liver biopsy features, as determined by Kaplan–Meier analysis, was presented in Fig. 4A and B C. There was a significantly different NLS according to BA-specific staging (*p* = 0.002). 83.3% (10/12) patients in BA-specific stage 4 died or underwent liver transplantation. The ROC curve analysis for predicting event occurrence based on three liver fibrosis staging indices Metavir, Ishak, and BA-specific (Fig. 4D). The AUC for Metavir was 0.708 (95% CI: 0.585–0.832, *p* = 0.003), for Ishak was 0.716 (95% CI: 0.594–0.838, *p* = 0.002), for BA-specific was 0.755 (95% CI: 0.639–0.872, *p* < 0.001).

The histological variables associated with NLS on univariate and multivariate COX regression were detailed in Table 3. A univariate COX regression analysis indicated that Ishak/Metavir (stage 4–6/3–4) (HR 3.194, 95% CI 1.227–8.312, *p* = 0.017), the BA-specific staging (stage 3–4) (HR 4.110, 95% CI 1.687–10.016, *p* = 0.002) and ductal proliferation (Severe) (HR 2.073, 95% CI 1.073–4.144, *p* = 0.039) were associated with mortality/liver transplantation events. However, none of the pathological characteristics could be used as an independent predictor of events in multivariate COX regression.

**Table 3:** Histological predictors for poor outcome

Variable	Hazard ratio	95% CI of HR	*p* value
**Univariate COX regression**			
Ishak/Metavir (4–6/3–4)	3.194	1.227–8.312	**0.017**
BA-specific (3–4)	4.110	1.687–10.016	**0.002**
Portal inflammation (Severe)	1.080	0.545–2.140	0.826
Intrahepatic cholestasis (Severe)	1.430	0.678–3.017	0.347
Ductal proliferation (Severe)	2.073	1.037–4.144	**0.039**
Age at PE (≥ 60)	1.646	0.817–3.315	0.163
**Multivariable COX regression**			
Ishak/Metavir (4–6/3–4)	0.558	0.064–4.844	0.597
BA-specific (3–4)	5.774	0.781–42.676	0.086
Ductal proliferation (Severe)	1.621	0.794–3.308	0.185

The COX proportional hazard regression model analysis includes histological characteristics (Ishak, Metavir, BA-specific stage and portal inflammation, intrahepatic cholestasis, ductal proliferation grade) and age at KP. Results with *p*-values < 0.05 were subjected to multivariate analysis after univariate analysis. *P*-values < 0.05 were considered statistically significant

### Factors associated with poor outcome in BA patients with mild liver fibrosis

**Fig. 5:**
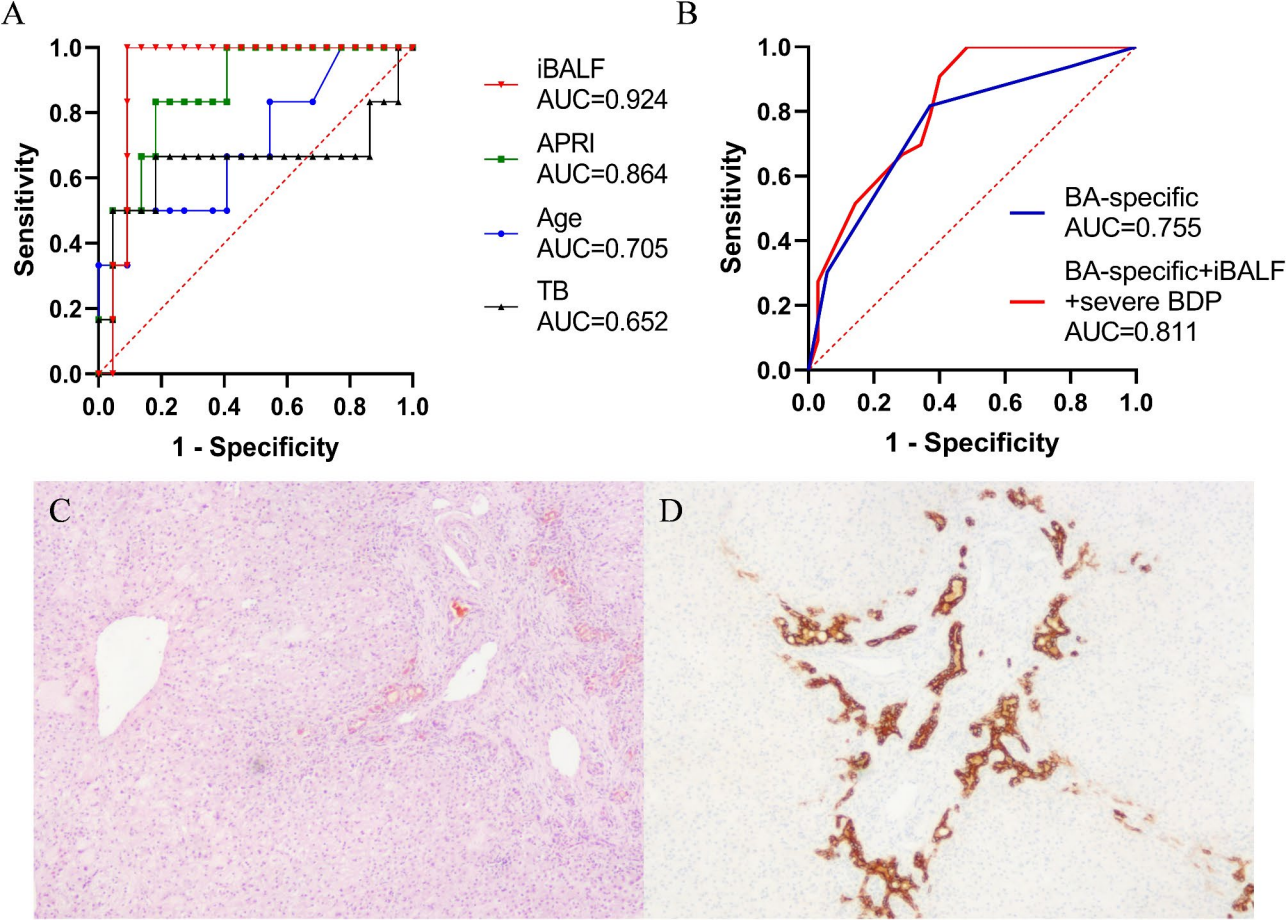
(**A**) The ROC curve for iBALF, APRI, TB and age with BA-specific stage 1–2. (**B**) The ROC curve of native liver survival for BA-specific staging alone, and BA-specific staging combined with iBALF and severe bile duct proliferation. A BA patient with BA-specific stage 2 had severe ductal proliferation. Fibrosis grading is stained for H&E (**C**) and bile duct proliferation is stained for CK19 (**D**) (40x total magnification)

A total of 28 BA patients graded 1–2 by the BA-specific staging had mild fibrosis, but 6 patients underwent death or liver transplantation events, with a median survival time of 5 months. A comparison of three pathological features in the good (NLS) and poor (death/liver transplantation) outcome groups revealed that severe BDP was significantly associated with prognosis (*p* = 0.038). Meanwhile, after comparing various laboratory indicators between the two groups, iBALF was able to best differentiate the prognosis of BA patients in stage 1–2 with an AUC of 0.924 (95% CI: 0.819-1.000, *p* = 0.0017) (Fig. 5A).

Furthermore, ROC curve analysis of all 68 BA patients for predicting prognosis showed that the AUC of BA-specific staging combined with iBALF > 4 (cut off value) and severe BDP (0.811, 95% CI: 0.710–0.913, *p* < 0.0001) was higher than BA-specific staging alone (0.755, 95% CI: 0.639–0.872, *p* < 0.001) (Fig. 5B). A BA patient with BA-specific stage 2, severe BDP and iBALF of 4.52 underwent liver transplantation at 6 months post-Kasai surgery (Fig. 5C, D).

## Discussion

This study presents the BA-specific liver fibrosis staging system, a reliable histological method suitable for BA patients, with the substantial inter-observer agreement and clear associations to clinical outcomes in BA.

BA has its specific histological features: fibrosis in the portal area, portal and periportal inflammatory responses, pathological hyperplasia of bile ducts, presence of bile infarcts, etc. Histological diagnosis of patients with BA is clinically important to identify those with worse prognoses. It is useful to use a scoring system to interpret histological changes, and some of the typical liver histological features of BA are associated with prognosis [24]. Previously, Shilpa Sharma [25] systemically analyzed the liver and portal histopathology of BA but was insufficient to precisely evaluate BA fibrosis. Hirofumi Tomita [26] developed a “BALF” score to assess the liver functions of BA but did not reflect on hepatic fibrosis. Therefore, it is worth considering whether the adult fibrosis staging systems now in use, Ishak and Metavir, are universally applicable in BA.

Since most patients with BA present with severe fibrosis or cirrhosis at first diagnosis, staging methods should subdivide them into prognostically meaningful categories. The BA-specific staging system was based on literature reports and the characteristic pathomorphological features of BA, which were classified into four stages of BA fibrosis. Ishak staging was detailed and suitable for studies that could better distinguish between fused necrosis and progressive hepatic sclerosis, but had the same score for different histopathological presentations, and was poor repeatability. Metavir staging was relatively simple and reproducible, but there was a breakpoint between stage 3–4 and a need for a consistent staging method. The BA-specific staging had good repeatability, with stage 3 as early cirrhosis and distinguishing it from stage 4 complete cirrhosis, but more sample validation was needed. This study compared the relationship between Ishak, Metavir, and BA-specific staging methods with the outcomes of BA patients after KP to select the most appropriate staging system for BA. Meanwhile, the typical intrahepatic histological features of BA associated with prognosis were observed, including portal inflammation, intrahepatic cholestasis, and ductal proliferation [27].

In the present study, different grades of liver fibrosis were observed in all 68 cases in the early stages of BA (62.5 ± 21.3 days). 41.2% of cases progressed to BA-specific stage 3, Metavir stage 3, or Ishak stage 4 at KP, which accounted for the largest proportion of cases. Thus, it was confirmed that the rapid progression of liver fibrosis and the high grades of fibrosis were in the early stage of BA. In addition, portal inflammation, intrahepatic cholestasis and ductal proliferation were observed in all cases of BA. With the progression of BA disease, the grades of portal inflammation and ductal proliferation increased significantly.

KP can relieve cholestasis and prolong the survival of patients with their liver. A pressing question for surgeons to answer is which type of patient is more likely to have a death/liver transplant after KP. Herein lies the strength of this study, which assessed the correlation between 3 fibrosis stages and other histopathological features with the prognosis after KP, allowing the selection of the optimal staging system. The effect of KP is usually judged by postoperative complete JC percentage and NLS rate [28]. It was observed that good or poor JC was significantly linked to liver fibrosis scores. The ROC curve analysis further revealed that the BA-specific stage had a greater AUC than the Ishak and Metavir stages, and thus a higher value for predicting JC. However, BA-specific staging was not found to be a possible predictor of JC in the multivariate regression analysis.

Even in patients who initially succeed in JC, the 2-year failure rate of disease progression is over 50% as the disease progresses [29]. The Kaplan-Meier curve and log-rank test result confirmed the significance of NLS between the four grades of the BA-specific staging. The predictive value of NLS was further analyzed with the ROC curve, and the BA-specific staging had a higher predictive value than Ishak and Metavir staging. The findings supported the notion of Weerasooriya et al. [10] that hepatic fibrosis, rather than age, was important for the NLS. Based on the above results, compared with the Ishak and Metavir, the BA-specific staging system has a better predictive value for outcomes after KP. In particular, 83.3% (10/12) patients in BA-specific stage 4 died or underwent liver transplantation. Therefore, BA specific stage 4 patients may require early liver transplantation treatment to a large extent.

However, even BA patients with the BA-specific staging assessment of mild fibrosis may have poor outcomes. Despite the early age of surgery and the mild degree of liver fibrosis, it did not mean that postoperative liver fibrosis had not developed rapidly and this rapidly progressive fibrosis was a risk factor for poor outcomes. After analyzing the pathological features and laboratory indicators of these patients, it was found that severe BDP and iBALF were associated with poor prognoses. BDP was known to have a prognostic value in BA, and an increase in BDP was associated with fibrosis progression in BA [21]. The preoperative iBALF score predicted the poor outcome of bile drainage surgery in BA patients and may be considered as a candidate for primary liver transplantation [30]. The AUC of prognosis for BA-specific staging combined with iBALF and severe BDP was higher than BA-specific staging alone, suggesting that combining these features would make it more appropriate for assessing BA prognosis. For patients with poor prognostic results, if Kasai surgery is performed, close follow-up and prompt symptomatic treatment are recommended after surgery.

This was a single-center retrospective study, with patients collected over a narrow 3 years window of time, so there were limitations in the sample size and the length of follow-up. Further, histology specimens may be subject to handling issues, harvesting issues and processing issues over the years, thus making relevant data collection difficult for some patients. Wedge liver biopsy may have limitations and tissue sampling errors in accurately representing BA intralobular pathologies. The proposed BA-specific staging system requires further prospective study to monitor longer NLS as a means of confirming its long-term prognostic relevance.

## Conclusions

This BA-specific staging system is capable of reflecting the true status of liver fibrosis in BA patients, and its combination with iBALF and severe BDP can well predict their prognosis. Furthermore, it provides value to clinicians in designing the appropriate surgical plan or treatment for BA patients.

## Data Availability

The datasets used and/or analysed during the current study are available from the corresponding author on reasonable request.

## Glossary

BA: Biliary atresia
KP: Kasai procedure
JC: Jaundice clearance
NLS: Native liver survival
LT: Liver transplantation
TB: Total bilirubin
DB: Direct bilirubin
AST: Aspartate aminotransferase
ALT: Alanine aminotransferase
GGT: γ-glutamyl-transpeptidase
ALP: Alkaline phosphatase
TBA: Total bile acid
BDP: Bile duct proliferation
ROC: Receiver operating characteristics
AUC: Area under the receiver operating characteristic curve
